# Hydrogel of Ketoconazole and PAMAM Dendrimers: Formulation and Antifungal Activity

**DOI:** 10.3390/molecules17044612

**Published:** 2012-04-18

**Authors:** Katarzyna Winnicka, Magdalena Wroblewska, Piotr Wieczorek, Pawel Tomasz Sacha, Elzbieta Tryniszewska

**Affiliations:** 1 Department of Pharmaceutical Technology, Faculty of Pharmacy, Medical University of Białystok, Mickiewicza 2c, 15-222 Białystok, Poland; Email: magdalena.wroblewska@umb.edu.pl; 2 Department of Microbiological Diagnostics, Faculty of Pharmacy, Medical University of Białystok, Kilińskiego 1, 15-089 Białystok, Poland; Email: piowie@umb.edu.pl (P.W.); sachpt@umb.edu.pl (P.T.S.); zdmik@umb.edu.pl (E.T.)

**Keywords:** PAMAM dendrimer, ketoconazole, hydrogel, antifungal activity, aqueous solubility

## Abstract

Ketoconazole (KET), an imidazole derivative with well-known antifungal properties, is lipophilic and practically insoluble in water, therefore its clinical use has some practical disadvantages. The aim of the present study was to investigate the influence of PAMAM-NH_2_ and PAMAM-OH dendrimers generation 2 and generation 3 on the solubility and antifungal activity of KET and to design and evaluate KET hydrogel with PAMAM dendrimers. It was shown that the surface charge of PAMAM dendrimers strongly affects their influence on the improvement of solubility and antifungal activity of KET. The MIC and MFC values obtained by broth dilution method indicate that PAMAM-NH_2_ dendrimers significantly (up to 16-fold) increased the antifungal activity of KET against *Candida* strains (e.g., in culture *Candida albicans* 1103059/11 MIC value was 0.008 μg/mL and 0.064 μg/mL, and MFC was 2 μg/mL and 32 μg/mL for KET in 10 mg/mL solution of PAMAM-NH_2_ G2 and pure KET, respectively). Antifungal activity of designed KET hydrogel with PAMAM-NH_2_ dendrimers measured by the plate diffusion method was definitely higher than pure KET hydrogel and than commercial available product. It was shown that the improvement of solubility and in the consequence the higher KET release from hydrogels seems to be a very significant factor affecting antifungal activity of KET in hydrogels containing PAMAM dendrimers.

## 1. Introduction

**Figure 1:**
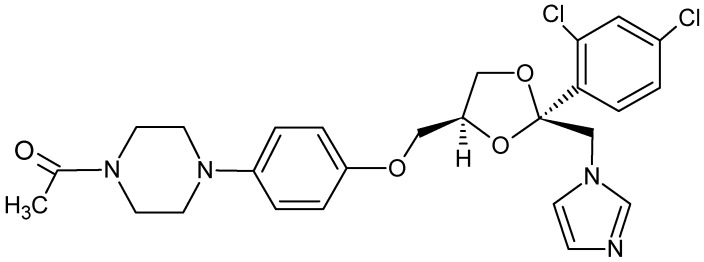
Chemical structure of the ketoconazole molecule.

Ketoconazole [KET, 1-acetyl-4-[4-[[(2RS,4SR)-2-(2,4-dichlorophenyl)-2-(1H-imidazol-1-ymethyl)- 1,3-dioxolan-4-yl]methoxy]phenyl]piperazine, Figure 1], is an imidazole antifungal agent, which is used both in the treatment of topical or systematic fungal infections [1,2,3]. It interferes with the fungal synthesis of ergosterol, a constituent of cell membrane specific for fungi [4]. KET also inhibits biosynthesis of triglycerides, phospholipids and oxidative or peroxidative enzyme activity, resulting in intracellular buildup of toxic concentrations of hydrogen peroxide [2]. Additionally, in the treatment of *Candida albicans* infections, it inhibits the transformation of blastospores to invasive mycelial forms [5]. However, ketoconazole is lipophilic and practically insoluble in water [1], therefore its clinical use has some practical disadvantages. 

**Figure 2:**
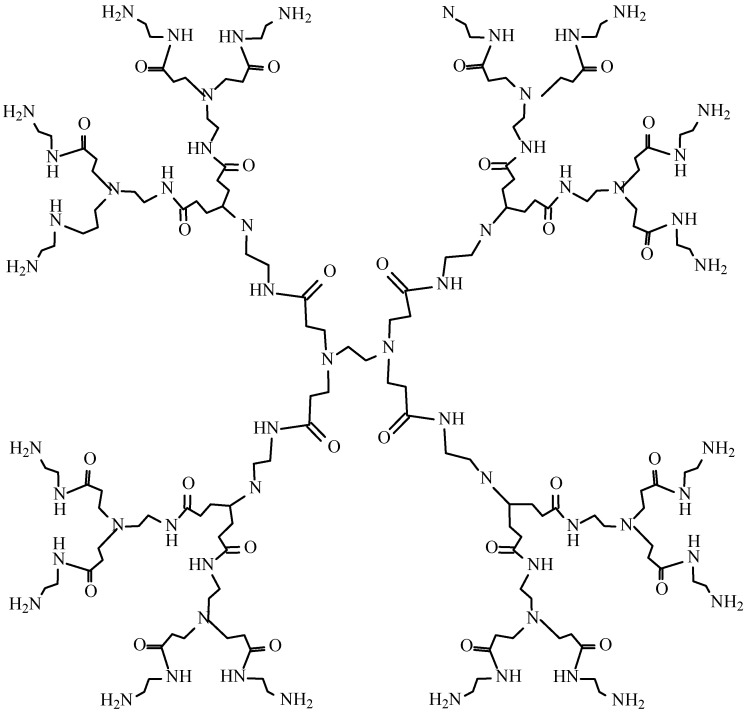
Chemical structure of PAMAM-NH_2_ dendrimer generation 2.

Recently, numerous efforts have focused on the development of drug carrier systems able to improve solubility and enhance the therapeutic efficacy of drugs [6]. Dendrimers are a relatively new, hyperbranched and monodisperse class of polymers, having defined molecular weight and host-guest entrapment properties [7,8,9,10]. They consist of a central core and three-dimensional branches with functionalized surface groups. Among dendrimers, amino acid-functionalized dendrimers, [poly(amidoamine), PAMAM] are the most popular (Figure 2). 

PAMAM dendrimers have demonstrated potential use as drug delivery systems. They are being considered as vehicles in several routes of administration, including oral, transdermal, and ocular or intravenous even [11]. Dendrimers might enhance the solubility of lipophilic drugs probably due to hydrophobic interactions, hydrogen bonding or electrostatic interaction between surface functional groups of the dendrimer and drug. Moreover, some of dendrimers show antibacterial and antifungal activity [12,13,14] and provide the opportunity for complex therapy in which the dendrimers are not only the drug carrier but also an adjunctive component of the dosage form [15]. So far PAMAM dendrimers have been mainly examined as vehicles for antibacterial agents, like quinolones, triazine-based antibiotics, or silver complexes [16,17,18]. Since, to our knowledge, there are no studies devoted to antifungal semisolid dosage forms with dendrimers, the goal of this work was to design hydrogels with KET and PAMAM dendrimers and to study the effect of PAMAM on their antifungal activity. 

## 2. Results and Discussion

### 2.1. Solubility Studies

KET is an imidazole derivative with broad-spectrum, fungistatic activity. However, its poor aqueous solubility requires addition of vehicles. Solubility is a physicochemical property of a substance, which influences absorption and action of drugs. One possibility to improve the solubility of lipophilic drugs is to combine these drugs with PAMAM dendrimers, which possess perfect solubility in a large number of solvents, particularly in water. Lipophilic cavities in PAMAM dendrimers and hydrophilic surface groups provide the availability to encapsulate or conjugate with many guest molecules. So far dendrimers have been employed for enhancing the solubility of more than 50 drugs [19]. 

**Figure 3:**
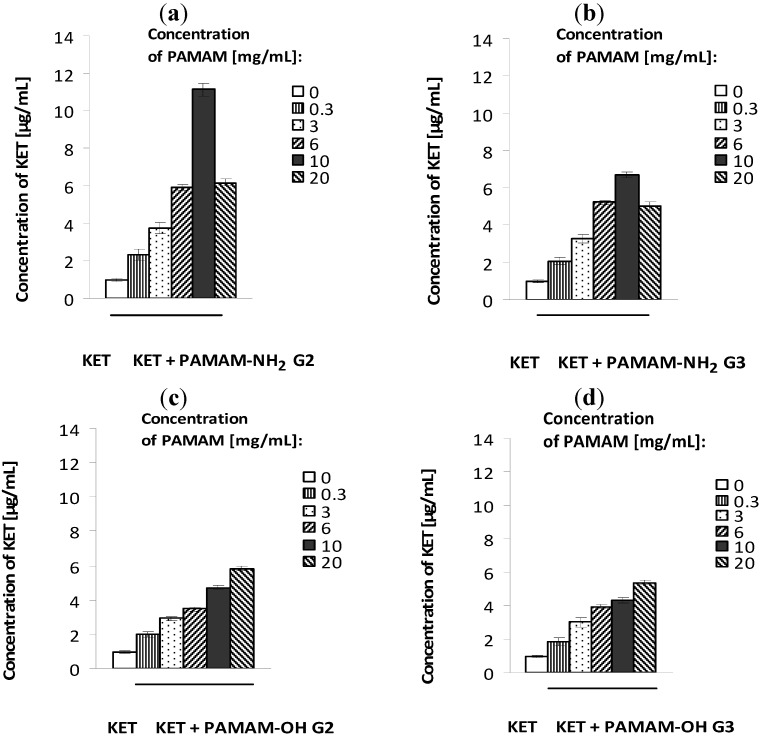
Solubility of ketoconazole in the presence of various concentrations of PAMAM-NH_2_ generation 2 (**a**), PAMAM-NH_2_ generation 3 (**b**), PAMAM-OH generation 2 (**c**), and PAMAM-OH generation 3 (**d**). Mean values from three independent experiments done in duplicate are presented.

The solubility study results are shown in Figure 3. All examined PAMAM dendrimers enhanced the solubility of KET, but PAMAM dendrimers with amine surface groups were significantly more potent. In the case of PAMAM-OH dendrimers, the solubility of KET increased linearly (from 0.98 μg/mL to 5.84 μg/mL) as a function of PAMAM concentration. Interestingly, among PAMAM-NH_2_ dendrimers generation 2 and generation 3 (G2 and G3), the highest increase in solubility was observed in the presence of PAMAM-NH_2_ G2 (from 0.98 μg/mL to 11.12 μg/mL). KET is a weak base (*pK_a_* = 6.54) [20], therefore it is rather encapsulated than attached to dendrimers’ amine surface groups and the main role in KET-PAMAM interactions might probably play hydrophobic interactions between dendrimer cavities and drugs or hydrogen bonds between tertiary amines within the PAMAM core and KET molecule. 

### 2.2. Influence of PAMAM on Antifungal Activity of KET

The results concerning the influence of PAMAM dendrimers on *in vitro* antifungal activity of KET obtained by broth dilution method are listed in Table 1 and Table 2. The MIC and MFC values indicate that PAMAM-NH_2_ dendrimers significantly increased the antifungal activity of KET against all studied *Candida* strains. The effect evoked by PAMAM-OH dendrimers was definitely weaker. KET in the presence of PAMAM-NH_2_ G2 was 2–16-fold more potent than pure KET (e.g., in culture *Candida albicans* 1103059/11 MIC value was 0.008 μg/mL and 0.064 μg/mL, and MFC was 2 μg/mL and 32 μg/mL for KET in solution of PAMAM-NH_2_ G2 and pure KET, respectively). Enhanced activity of KET could not be contributed to the dendrimer itself as PAMAM-NH_2_ or PAMAM-OH G2 and G3 in the studied concentration range (from 10 mg/mL to 0.000008 mg/mL) did not arrest the growth of the fungi. In all studied *Candida* species incubated for 48 h with PAMAM dendrimers, growth of yeast cells was observed.

**Table 1:** Minimum Inhibitory Concentration—MIC (in μg/mL) of ketoconazole in the presence of PAMAM-OH and PAMAM-NH_2_ dendrimers generation 2 (G2) and generation 3 (G3).

Fungal species	MIC (μg/mL)				
	KET	KET + PAMAM-OH		KET + PAMAM-NH_2_	
		G2	G3	G2	G3
*Candida albicans* 1103055/11	0.064	0.032	0.032	0.016	0.016
*Candida albicans* 1103059/11	0.064	0.032	0.032	0.008	0.016
*Candida krusei* 1103055/11	0.064	0.064	0.064	0.064	0.032
*Candida glabrata* 1102890/11	0.064	0.064	0.032	0.016	0.032
*Candida dubliniensis* 1103124/11	0.016	0.016	0.016	0.004	0.016
*Candida parapsilosis* ATCC 22019	0.016	0.016	0.008	0.008	0.008

**Table 2:** Minimum Fungicidal Concentration—MFC (in μg/mL) of ketoconazole in the presence of PAMAM-OH and PAMAM-NH_2_ dendrimers generation 2 (G2) and generation 3 (G3).

Fungal species	MFC (μg/mL)				
	KET	KET + PAMAM-OH		KET + PAMAM-NH_2_	
		G2	G3	G2	G3
*Candida albicans* 1103055/11	2	2	2	0.5	1
*Candida albicans* 1103059/11	32	32	16	2	8
*Candida krusei* 1103055/11	0.25	0.25	0.25	0.25	0.25
*Candida glabrata* 1102890/11	64	64	64	4	16
*Candida dubliniensis* 1103124/11	1	0.125	0.25	0.5	0.125
*Candida parapsilosis* ATCC 22019	0.032	0.032	0.032	0.016	0.016

This enhanced antifungal activity of KET by PAMAM-NH_2_ dendrimers might be explained as the result of a binding reaction between protonated dendrimer amino groups with negatively charged fungal cell surface molecules [21]. However, if this were the only operative mechanism of action, higher generations of PAMAM should be expected to demonstrate stronger antifungal activity as they possess more amino groups available for such reactions (16 amino groups in G2 versus 32 amino groups in G3 of PAMAM). Therefore, it should also be noted, that dendrimers might enhance the cellular delivery of drugs and the size of the dendrimer molecule probably affects its ability to penetrate the cell membrane and in the consequence antimicrobial activity of encapsulated drugs. In this study, PAMAM-NH_2_ G2 with a smaller molecule (2.0 nm in diameter) was found to be more active than PAMAM-NH_2_ G3 (3.1 nm). Similar effects was observed when carbosilane dendrimers were studied as antimicrobial agents [22]. Venuganti *et al*. using fluorescent labelled dendrimers has also shown that lower generation of dendrimers penetrated to a greater extent than higher generations [23].

### 2.3. Antifungal Activity of Hydrogels with KET and PAMAM Dendrimers

Treatment of skin infections requires proper topical dosage forms, which provide high concentrations of the drug in the target site for therapeutic activity. In the case of superficial fungal skin infections, in which the main location of the pathogen is the epidermis, clinical efficacy of the therapy depends on the drug’s ability to penetrate into the stratum corneum to inhibit the fungus growth [24,25]. As skin is negatively charged at physiological pH [26], cationic dendrimers are expected to interact with biological membranes and increase skin permeability [23]. Studies performed with confocal laser scanning microscopy showed a higher skin permeation of fluorescent labeled cationic dendrimers, while other dendrimers showed a lower skin permeation [23]. Gardikis *et al*. have found that dendrimers interact with hydrophilic head groups of phospholipids and fluidize the lipid membranes [27]. Although phospholipids are not a component of the skin lipid matrix, dendrimers can affect the polar head groups of skin ceramides and free fatty acids. Additionally, Perumal *et al*. have shown that dendrimers serve as a carrier and transport the lipophilic drug in the solubilised form to the skin surface from where it partitions into the stratum corneum due to the high affinity for skin lipids [28]. Moreover, dendrimers have been shown to be useful in topical delivery of nonsteroidal anti-inflammatory drugs, antiviral, or anticancer drugs and it was found that PAMAM dendrimer-drug formulations showed increased transdermal availability [29]. Hence, hydrogel formulations containing PAMAM-NH_2_ dendrimers appeared to be a viable approach for future topical delivery of drugs. Therefore, in the next step hydrogel formulations with KET and PAMAM-NH_2_ dendrimers were prepared (Table 3) and their antifungal activity by agar diffusion method in comparison to formulations without PAMAM was evaluated. As a control, solution of KET in DMSO, commercially available topical cream (Nizoral^®^), hydrogels with PAMAM-NH_2_ dendrimers at concentration 3 mg/mL and 6 mg/mL, and placebo hydrogel was used. The examined hydrogel formulations with PAMAM-NH_2_ dendrimers did not exert significant activity against tested *Candida* strains. The values of zone inhibition after 24 h of incubation at 37 °C ± 0.1 °C were found to be below 5.8 mm (data not shown) and were similar to that obtained for placebo formulation H0 (Table 4). The values of zone inhibition in *Candidaalbicans* 1103055/11 culture produced by KET standard, Nizoral^®^, placebo formulation (H0), KET-hydrogel (H1), KET-PAMAM NH_2_ G2 and G3 hydrogels (H2–H5) were 25.6 ± 0.1 mm, 15.0 ± 0.2 mm, 5.0 ± 0.1 mm, 16.2 ± 0.3 mm, 18.3 ± 0.4 mm, 18.8 ± 0.5 mm, 16.9 ± 0.4 mm, and 17.3 ± 0.2 mm, respectively (n = 3) (Table 4). Although the antifungal potency of KET-PAMAM hydrogels differs according to the *Candida* species evaluated, their enhanced antifungal effect has been demonstrated (Table 4). Interestingly, under that condition KET-hydrogels with PAMAM-NH_2_ G2 and G3 showed similar mean diameter of zone of inhibition. It might be caused by similar diffusion process of PAMAM-NH_2_ G2 and G3 from viscous hydrogels. Moreover, it should be noted, that the antifungal activity of KET in hydrogels might be influenced by excipients used. It was found that *in vitro* antifungal activity of fluconazole was reduced by the presence of propylene glycol in the formulation [25]. As propylene glycol is a frequently used solvent and skin penetration enhancer and it is a component of commercially available Nizoral^®^ cream, it was also used as an excipient to obtain hydrogels (Table 3). To sum up, it is evident that designed KET-hydrogels with PAMAM-NH_2_ were more efficient than hydrogels with pure KET and than marketed product (Table 4). The higher antifungal activity of KET-PAMAM hydrogels might be due to an increased solubility of KET (Figure 3) and electrostatic interactions with negatively charged fungi cells surface molecules [21].

**Table 3:** Formulation of prepared hydrogels.

Ingredient (g)	Formulation code					
	H0	H1	H2	H3	H4	H5
**KET**	-	2.0	2.0	2.0	2.0	2.0
**Carbopol 980**	0.4	0.4	0.4	0.4	0.4	0.4
**20% NaOH**	q.s.	q.s.	q.s.	q.s	q.s.	q.s
**Propylene glycol**	10.0	10.0	10.0	10.0	10.0	10.0
**Tween 80**	1.0	1.0	1.0	1.0	1.0	1.0
**Bronopol**	0.01	0.01	0.01	0.01	0.01	0.01
**PAMAM-NH_2_ G2**	-	-	0.3	0.6	-	-
**PAMAM-NH_2_ G3**	-	-	-	-	0.3	0.6
**Purified water (up to)**	100.0	100.0	100.0	100.0	100.0	100.0

**Table 4:** Antifungal activity of prepared hydrogels in comparison to reference standard.

Fungal species	Zone of inhibition (mm)							
	KET	Nizoral	H0	H1	H2	H3	H4	H5
*C. albicans* 1103055/11	25.6 ± 0.1	15.0 ± 0.2	5.0 ± 0.1	16.2 ± 0.3	18.3 ± 0.4	18.8 ± 0.5	16.9 ± 0.4	17.3 ± 0.2
*C. albicans* 1103059/11	24.1 ± 0.3	14.4 ± 0.4	4.5 ± 0.2	12.2 ± 0.2	17.3 ± 0.3	17.4 ± 0.4	16.4 ± 0.5	18.5 ± 0.4
*C. glabrata* 1102890/11	26.4 ± 0.1	16.0 ± 0.5	5.0 ± 0.2	21.9 ± 0.2	25.3 ± 0.4	20.4 ± 0.4	23.5 ± 0.5	19.7 ± 0.3
*C. dubliniensis* 1103124/11	24.4 ± 0.4	15.0 ± 0.3	5.2 ± 0.3	17.7 ± 0.5	20.4 ± 0.2	19.9 ± 0.3	19.3 ± 0.3	16.4 ± 0.4
*C. parapsilosis* ATCC 22019	48.4 ± 0.3	33.0 ± 0.4	5.0 ± 0.1	35.0 ± 0.6	38.9 ± 0.2	31.8 ± 0.5	37.7 ± 0.3	37.0 ± 0.2

### 2.4. *In Vitro* Release of KET

**Figure 4:**
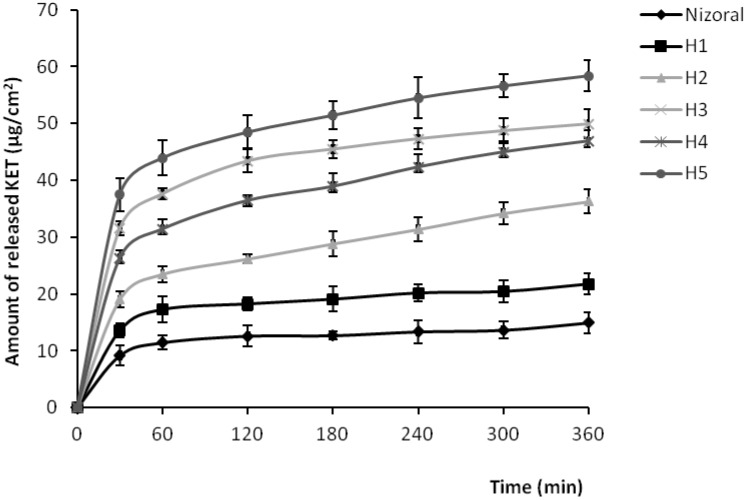
Amount of KET per unit area (μg/cm^2^) released from different hydrogel formulations and commercially available product.

Results of the drug release evaluation are shown in Figure 4. The release profile was followed for 6 h and it is clearly visible that KET was definitely faster released from formulations containing PAMAM dendrimers. After 6 h, the cumulative amount of KET released from hydrogels with PAMAM G2 at concentration 3 mg/mL and 6 mg/mL (formulations H2 and H3), from hydrogels with PAMAM G3 3 mg/mL and 6 mg/mL (formulations H4 and H5), and from KET hydrogel without PAMAM (formulation H1) was found to be 36 ± 4 μg/cm^2^, 50 ± 3 μg/cm^2^, 47 ± 2 μg/cm^2^, 58 ± 5 μg/cm^2^ and 21 ± 3 μg/cm^2^, respectively. For comparison, after 6 h of the study, the amount of KET released from commercially available product was only 14 ± 3 μg/cm^2^. The amount of released KET increased with increasing concentrations of PAMAM. This might be due to improved solubility of KET with increasing concentrations of PAMAM dendrimers (Figure 3), which allows the presentation of the drug to the receptor medium in a more diffusible form. Important to note is that the release of KET was also dependent on the size of the dendrimer molecule with generation 3 being the more potent. Therefore, beside the enhancing cellular delivery of drugs, and penetration through the cell membranes [22,23], the improvement of solubility and in the consequence the higher KET release from hydrogels seems to be a very significant factor affecting antifungal activity of KET in hydrogels containing PAMAM dendrimers.

Using dendrimers as drug carriers for topical delivery might be beneficial as they only produces a transient effect without skin irritation, therefore *in vivo* toxicity studies in rats are underway and will be described in a due course. However, it was stated earlier, that monomers penetrated deeper inside the skin to the dermis and caused skin irritation, whereas polymers did not cause irritation, because were retained in the stratum corneum [30,31]. As dendrimers combine features of small organic molecules and polymers, their applications in drug delivery systems might make them reliable and safe alternatives to traditional compounds.

## 3. Experimental

### 3.1. Materials

Ketoconazole was a gift from Polfarmex S.A. (Kutno, Poland). PAMAM dendrimers generation 2 (G2) and generation 3 (G3) with amine and hydroxyl surface groups, RPMI 1640 medium, dimethyl sulfoxide (DMSO), 2-bromo-2 nitropropane-1,3-diol (bronopol), and Tween 80 were provided by Sigma Aldrich (St. Louis, MO, USA), as were most other chemicals and buffers used. Stock cultures of *C. parapsilosis* ATCC 22019 were purchased from Microbiologics (St. Cloud, MN, USA). Methanol and acetonitril HPLC grade were obtained from Merck (Darmstadt, Germany). HA membrane filters (0.45 µm) were received from Millipore (Billerica, MA, USA) and Cuprophan^®^ from Medicell (London, UK). Carbopol 980 was purchased from Lubrizol (Cleveland, OH, USA), propylene glycol was from Chempur (Piekary Slaskie, Poland). Nizoral^®^ cream (20 mg/g) was a product of Janssen-Cilag (Beerse, Belgium).

### 3.2. Solubility Studies

The solubility of KET was determined by using shake-flask method. Briefly, an excess amount of KET was added to each vial containing 5 mL of the selected vehicle (water or 0.3 mg/mL, 3 mg/mL, 6 mg/mL, 10 mg/mL, and 20 mg/mL solutions of G2 and G3 PAMAM dendrimers with amine or hydroxyl surface groups). Mixtures were mechanically shaken for 48 h at 25 °C ± 0.5 °C and allowed to stand for 24 h to attain equilibrium. Then mixtures were centrifuged at 3,000 rpm for 15 min, followed by filtration through HA membrane filter (0.45 μm), diluted appropriately with the methanol and analyzed by HPLC method at 231 nm against a standard [32].

### 3.3. HPLC Analysis

The solubility of KET was determined on an Agilent Technologies 1200 HPLC system equipped with a G1312A binary pump, a G1316A thermostat, a G1379B degasser and a G1315B diode array detector (Agilent, Waldbronn, Germany). Data collection and analysis were performed using Chemstation 6.0 software. Isocratic separation was achieved on a Zorbax Eclipse XDB-C18, 4.6 × 150 mm, 5 μm column (Agilent, Waldbronn, Germany). Mobile phase was methanol-acetonitrile-phosphate buffer pH 6.8 (35:40:25, v/v), the flow rate was 1.0 mL/min and UV detection was performed at a wavelength of 231 nm [32]. The column temperature was maintained at 25 °C. For injection into the HPLC system 20 µL of sample was used. All reagents used for analysis were HPLC grade. The retention time of ketoconazole was 4.0 min. The standard calibration curve was linear over the range of 1–100 μg/mL.

### 3.4. Test Organisms

The antifungal activity was evaluated against yeast cultures *Candida parapsilosis* ATCC 22019, and against susceptible and drug resistant clinical strains belonging to the species *C. albicans*, *C. glabrata*, *C. krusei* and *C. dubliniensis*. *Candida* strains were isolated from selected patients with candidiasis, identified morphologically [33], and stored in potato dextrose broth at −70 °C. Prior to antifungal susceptibility testing, each isolate was inoculated on potato dextrose agar plates to ensure optimal growth characteristics and purity. Then yeast cells were suspended in saline and adjusted spectrophotometrically to RPMI 1640 medium. 

### 3.5. Antifungal Agent

KET > 99% pure was obtained from Polfarmex S.A. (Kutno, Poland) as a fluffy white powder. KET is insoluble in water but dissolves in organic solvents [1]. A stock solution of 5,120 μg/mL was prepared by dissolving KET in sterile DMSO and in 1 mL (10 mg/mL) PAMAM dendrimers G2 and G3 with amine or hydroxyl surface groups. These solutions were stored in dark glass bottles and were used for all experiments in this study. Series of double diluting solutions of above compounds were prepared in RPMI 1640 medium [with L-glutamine, without sodium bicarbonate and buffered to pH 7.0 with 3-(*n*-morpholino)propanesulfonic acid] obtaining the final concentration of KET in the range from 512 μg/mL to 0.004 μg/mL. Solutions of PAMAM dendrimers and DMSO were also evaluated in the absence of KET. 

### 3.6. Broth Microdilution Method

Antifungal activity was tested by using broth microdilution method according to the Clinical and Laboratory Standards Institute (CLSI) guidelines [34,35]. The medium used for susceptibility testing was RPMI 1640 with L-glutamine, without sodium bicarbonate and buffered to pH 7.0 with 3-(*n*-morpholino)propanesulfonic acid. The initial density of *Candida* was approximately 2–5 × 10^6^ colony forming units (CFU)/mL. Inoculums of fungi (density of 0.5 in McFarland scale) were prepared in sterile solution of 0.9% NaCl solution. Then tested strains were suspended in RPMI 1640 medium to give a final density of 5 × 10^4^ CFU/mL. Solutions of PAMAM, KET, KET in PAMAM dendrimers and suspensions of fungi were inoculated onto microtiter plates. The growth control, sterility control and control of antifungal compounds were used. Plates were incubated under normal atmospheric conditions at 35 °C for 48 h, and next minimum inhibitory concentration (MIC) values have been determined. The MIC was defined as the lowest concentration required to arrest the growth of the fungi. For determination of minimum fungicidal concentration (MFC), a 0.01 mL aliquot of the medium drawn from the culture tubes showing no macroscopic growth at the end of the 24 h culture was subcultured on potato dextrose agar plates to determine the number of vital organisms and incubated further for appearance of yeast-like growth. The MFC was defined as the lowest concentration of the agent at which no colonies are observed [36]. All MIC and MFC experiments were repeated three times. The stock solution of pure KET was used as positive control. Solvent and media controls were used for reference.

### 3.7. Preparation of Hydrogels

The hydrogels were prepared by dissolving bronopol in purified water and then the Carbopol 980 was gradually added and steered by an automatic steering machine until homogenous mixture appeared. Next mixture was neutralized (to pH 6.0) by dropwise addition of 20% solution of sodium hydroxide to allow gel formation. Mixing was continued until a transparent gel was received and after that other ingredients were added (Table 3). The obtaining gels were homogenized for 30 min and stored at room temperature for 24 h before use. 

### 3.8. Plate Diffusion Method

For the investigation of the antifungal activity of hydrogels, the plate diffusion method was used. Petri dishes containing Sabouraud’s dextrose agar were seeded with 100 µL of the fungal inoculum. The plates were dried at room temperature for 15 min, then 5 mm diameters wells were cut in the inoculated agar plates and 100 mg of various formulations were placed into each well. Hydrogel without KET and PAMAM dendrimers (placebo), hydrogel with PAMAM-NH_2_ dendrimers at concentration 3 mg/mL and 6 mg/mL, hydrogel with KET, 2% solution of KET in DMSO (reference standard) and commercial available cream were used as a control. The plates were incubated at 37 °C ± 0.1 °C for 24 h. Antifungal activity was expressed as the mean of inhibition zones (mm) around each well with an accuracy of 0.1 mm [37]. All determinations were made in triplicate for each test microorganism. 

### 3.9. *In Vitro* Release of KET

*In vitro* release of KET from prepared hydrogels and commercially available product was measured through natural cellulose membrane (Cuprophan, Medicell, London, UK) using an Enhancer cell (Agilent Technologies, Cary, NC, USA) with surface area of 3.80 cm^2^. The enhancer cell used in this study consisted of a Teflon load ring, a cap, a membrane, and a drug reservoir. About 3.0 g of each formulation (“infinite dose”) was placed in the drug reservoir on the top of the membrane making certain that no entrapped air was present at the interface of the gel and the membrane. A USP Apparatus 2, Dissolution Tester (Agilent 708-DS, Agilent Technologies, Cary, NC, USA) with mini vessels (250 mL) and mini paddles with a rotating speed of 75 rpm was used to measure the release of KET from the enhancer cell assembly. The receptor compartment was filled with 100 mL of acetic buffer (pH 5.5) with 1% SDS to provide the *sink* conditions and maintained at 32 °C ± 0.5 °C. At the predetermined time intervals (0.5, 1, 2, 3, 4, 5, and 6 h), 1 mL aliquots were removed and replaced with an equivalent amount of fresh buffer. The drug content in the withdrawn samples was determined by HPLC method as described earlier. All release experiments were conducted in triplicate.

### 3.10. Data analysis

The results were analysed by analysis of variance (ANOVA) and multiple comparison were done to check statistical significance. The statistical significance between means was verified by Sheffe’s comparison test accepting *p* < 0.05 as significant.

## 4. Conclusions

The surface charge of PAMAM dendrimers strongly affects their influence on the improvement of solubility and antifungal activity of KET. It was shown that using PAMAM-NH_2_ dendrimers not only improve the solubility and the *in vitro* release of KET, but also enhance the antifungal activity of KET in prepared hydrogels. This study reveals that PAMAM dendrimers seem to be promising vehicles which can be used for drug delivery in topical fungal infections.
